# Are housing circumstances associated with faster epigenetic ageing?

**DOI:** 10.1136/jech-2023-220523

**Published:** 2023-10-10

**Authors:** Amy Clair, Emma Baker, Meena Kumari

**Affiliations:** 1 Australian Centre for Housing Research, The University of Adelaide, Adelaide, South Australia, Australia; 2 Institute for Social and Economic Research, University of Essex, Colchester, UK

**Keywords:** housing, genetics, health inequalities, policy, ageing

## Abstract

**Background:**

Numerous aspects of housing are associated with health. However, the pathways between housing and health, particularly the psychosocial elements of housing, are less well understood. Epigenetic information alongside social survey data offers an opportunity to explore biological ageing, measured using DNA methylation, as a potential pathway through which housing affects health.

**Methods:**

We use data on housing and DNA methylation from the UK Household Longitudinal Study, linked with prior survey responses from the British Household Panel Survey, covering adults in Great Britain. We explore the association between epigenetic ageing and housing circumstances, both contemporary and historical, using hierarchical regression.

**Results:**

We find that living in a privately rented home is related to faster biological ageing. Importantly, the impact of private renting (coefficient (SE) 0.046 years (0.011) vs owned outright, p<0.001) is greater than the impact of experiencing unemployment (coefficient 0.027 years (0.012) vs employed, p<0.05) or being a former smoker (coefficient 0.021 years (0.005) vs never smoker, p<0.001). When we include historical housing circumstances in the analysis, we find that repeated housing arrears and exposure to pollution/environmental problems are also associated with faster biological ageing.

**Conclusion:**

Our results suggest that challenging housing circumstances negatively affect health through faster biological ageing. However, biological ageing is reversible, highlighting the significant potential for housing policy changes to improve health.

**WHAT IS ALREADY KNOWN ON THIS TOPIC**

- Links between housing circumstances and aspects of health are well established.
- What is less well understood are the pathways between the two.

**WHAT THIS STUDY ADDS**

- Housing circumstances—specifically private renting, repeated housing arrears and exposure to pollution—are associated with faster biological ageing.

**HOW THIS STUDY MIGHT AFFECT RESEARCH, PRACTICE OR POLICY**

- Our findings demonstrate the potential epigenetic impacts of housing.
- Compared with other social determinants, such as unemployment, housing problems are associated with a greater impact on ageing, indicating that improving housing should be a target of health interventions.

## Introduction

Housing is an important social determinant of health.^1^ Numerous aspects of housing, tangible and intangible, are linked with health, both physical and mental. New health indicators linked with social survey data offer the opportunity to expand our understanding of the relationship between housing and health. Recently, DNA methylation measures of biological ageing have been proposed as a means to understand the mechanisms that underpin social differences in health. In this paper, we explore whether a person’s housing circumstances are related to biological ageing.

### Estimating biological age(ing)

There is a significant variation in the health of people of the same chronological age.^2^ Comparison between biological and chronological age has the potential to shed light on health differences and their causes, with particular relevance to understanding the social determinants of health. Since at least the 1980s, the potential for a biomarker of biological age or biological pace of ageing has been proposed.^3 4^

DNA methylation-based measures have been suggested as the most promising indicators of biological ageing in terms of validity and predictive power.^3 4^ DNA methylation is an epigenetic mechanism that occurs when a methyl group attaches onto DNA at the C5 position of the cytosine-forming 5-methylcytosine, potentially affecting gene expression.^5^ Because DNA methylation changes with age, methylation is regarded as ‘a fundamental mechanism that drives human ageing’.^2^ Using algorithm-derived weighted averages of methylation levels at different CpG sites, the so-called ‘epigenetic clocks’ produce estimates of biological age, called DNA methylation age (DNAmAge).^3 6 7^ Research using these measures shows they have strong links with mortality^8^ and morbidity.^4^ In particular, positive age acceleration is associated with disease and death.^2 3 6 9^

Previous work has examined the ability of epigenetic measures of biological ageing to predict mortality or disease^2^ independent of chronological age. An emerging body of work uses the epigenetic measures as an outcome rather than a predictor to explore whether social factors and individual behaviours affect biological ageing (for example see previous work^7^). This work has linked accelerated ageing with a number of social and structural factors, including cumulative lifetime stress, income, education,^3^ employment status, socioeconomic position and mobility,^10 11^ and financial pressure.^12^ DNAmAge measures may therefore help to explain the mechanisms underlying the relationship between housing and health, particularly given that housing circumstances are themselves indicators of socioeconomic position,^13^ although not typically included in indicators of socioeconomic position in DNAmAge analyses.^11^

### Housing and health

Concerns about the impacts of housing on health have a long history (for example see previous works^14 15^). Early work was typically concerned with the physical conditions of housing, such as crowding and cold, but over time research increasingly came to include the non-tangible aspects of housing, such as affordability and security.^1^ More recently, research investigating the links between housing and health has emphasised the overlapping and intertwined nature of housing issues^16 17^ and the way that these may influence health.

Capturing overlapping aspects of housing can be challenging, but housing tenure is a potentially effective latent variable, at least in countries such as the UK that have highly distinct tenures in terms of policy treatment. Housing tenure refers to the legal means in which a person or household inhabits their home. In the UK, there are three main tenures, each representing roughly one-third of English households (in 2011/2012): outright ownership, mortgaged ownership and rental. Within the rental tenure, approximately half rent from a private landlord (private rent) and half from a local housing authority or association (social rent).^18^ Other tenure types include shared ownership and rent-free accommodation.

Housing policies, particularly regarding enforcement of housing standards, financial support, and security of tenure, mean that people’s housing experiences vary considerably depending on their housing tenure. Across three of the four aspects of housing precariousness^17^—affordability, security (both of which broadly relate to the psychosocial qualities of housing) and quality (which broadly relates to the physical/material characteristics of housing)—private renting in the UK compares considerably worse than other tenures. Private renting is highly insecure, average quality is lower, and costs are highest—both in terms of absolute rents and rents as a proportion of income (although it should be noted that conditions for private renters in Scotland and Wales are likely improved following changes to standard tenancies that occurred after the data used here were collected).

There are many potential causal mechanisms through which the material and psychosocial characteristics of housing may influence health and therefore biological ageing. Summaries are available elsewhere,^19 20^ but briefly, physical housing conditions are linked to health via exposure to things such as cold, mould, crowding and injury hazards, while psychosocial aspects of housing are linked to health via stress and stigma.

## Methods

### Data

The UK Household Longitudinal Study (UKHLS) is a longitudinal panel survey which covers a representative sample of approximately 40 000 UK households. It began in 2009 when it replaced the British Household Panel Survey (BHPS) which had been running since 1991. The UKHLS began with a new sample, absorbing the BHPS sample in wave 2. Extensive efforts have been made to harmonise the BHPS and UKHLS datasets, enabling linkage across datasets. Both the BHPS and the UKHLS contain considerable information about individuals and households, including housing details. Between 2010 and 2012 (corresponding to waves 2 and 3 of the UKHLS), separate nurse visits were conducted to collect additional health information including blood samples from survey respondents, approximately 5 months after main UKHLS survey response. Logistical issues meant that Northern Ireland was not included in the nurse visit component.^21^ Blood samples were analysed resulting in a number of health indicators, including six measures of biological age calculated by the UKHLS team from DNA methylation data.^22 23^

Given our interest in both historical and contemporary housing experiences on health, our sample is the 1420 survey respondents originally from the BHPS sample for whom blood samples and methylation data are available. Their methylation data are linked to the corresponding UKHLS survey (wave 3) and survey responses from BHPS waves 9–18 (years 1999–2008).^21^ Eligibility for blood sampling was based on their full participation in the corresponding UKHLS survey and respondents were required to be over the age of 16 years, have completed the UKHLS survey in English and not be pregnant. Of those eligible for blood sample collection from the BHPS sample, 56.6% took part. DNA methylation of collected blood samples was restricted to those of white ethnicity.

### Measuring biological age(ing)

Many different approaches to DNAmAge have been developed. These can be split into first-generation approaches (often called clocks) and second-generation approaches. First-generation clocks such as the Horvath,^24^ Hannum *et al*,^25^ and Lin *et al*’s^26 27^ are based on prediction of chronological age. However, accurate prediction of chronological age is not necessarily the main goal of many analyses.^9^ Second-generation measures, including the DNAm PhenoAge^28^ and DunedinPoAm,^6^ are instead trained on clinical biomarkers in order to predict phenotypic, rather than chronological, age. In particular, the DunedinPoAm clock is designed to indicate a person’s pace of ageing—to calculate ‘rate’ of ageing rather than ‘state’ of ageing. Those deemed to be ageing faster using DunedinPoAm have been found to have worse physical and cognitive functioning, as well as other indicators of ageing, 7 years after DNA data collection.^6^

Given our interest in whether housing difficulties potentially cause people to biologically age faster than those not experiencing housing difficulties, the potentially transient nature of such effects^29^ as well as the cross-sectional nature of our DNA methylation measure, we focus on the DunedinPoAm measure. DunedinPoAm also appears to be associated with subjective health to a greater extent than alternative epigenetic measures, but retains the link with objective measures such as mortality and morbidity.^6^

DNA was extracted from UKHLS blood samples and DNA methylation was measured using the Illumina Infinium HumanMethylationEPIC BeadChip kit (Illumina, San Diego, California, USA). The DunedinPoAm algorithm was calculated from these data as previously described in Bao *et al*.^10^

### Predictor variables

As in Clair and Hughes,^19^ we consider all possible housing elements available in the data to reflect the varied and complex role housing plays in people’s lives. We include both material elements of housing: tenure, building type, receipt of housing benefit (financial support available to renters), presence of central heating (as a proxy for adequate warmth which is not asked in wave 3 of the UKHLS), whether the home is an urban or rural area; and psychosocial elements: housing cost burden, payment arrears, an indicator of overcrowding, and an indicator of moving expectations and preferences. Unfortunately, the UKHLS does not include any variables relating to the physical qualities of the home that are suitable for this analysis; however, some were included in the BHPS questionnaires and are included in our historical analysis. Variables relating to contemporary housing circumstances and their weighted proportions are shown in table 1, and those relating to historical housing circumstances in table 2.

**Table 1:** Weighted proportions, contemporary housing variables, complete cases

Variable	Categories	Proportions
Tenure	Owned outright	0.403
	Owned with mortgage	0.387
	Social rent	0.138
	Private rent	0.069
	Other	0.003
Building type	Detached	0.303
	Semi-detached	0.340
	Terrace	0.263
	Flat	0.090
	Other	0.003
Receive housing benefit	No	0.964
	Yes	0.036
Has central heating	Yes	0.954
	No	0.046
Rural/urban	Urban	0.723
	Rural	0.277
Housing cost burden (spend over 1/3 of income on housing, income below median)	No	0.973
	Yes	0.027
Housing payment arrears (2+ months late with payment)	No	0.951
	Yes	0.050
Overcrowded (less than one bedroom per person/couple)	No	0.949
	Yes	0.051
Moving desires and expectations	Want to stay, expect to stay	0.673
	Want to move, expect to move	0.069
	Want to stay, expect to move	0.020
	Want to move, expect to stay	0.237

Values may not sum to 1 due to rounding.

**Table 2:** Historical housing circumstances from final 10 years of BHPS survey, proportions, complete cases

		Weighted proportion experiencing issue at some point
Does your accommodation have any of the following problems?	Damp walls, floors, foundation, etc	0.286
	Shortage of space	0.559
	Noise from neighbours	0.390
	Other street noise	0.489
	Too dark, not enough light	0.235
	Lack of adequate heating facilities	0.193
	Condensation	0.397
	Leaky roof	0.219
	Rot in window frames or floors	0.305
	Pollution, grime or other environmental problems caused by traffic or industry	0.260
Reported experiencing…	Housing payment difficulties (problems paying for housing for over 1 year)	0.249
	Housing payment arrears	0.069
	Moving desires mismatch*	0.654
	No central heating	0.207

*Reported wanting to move but expecting to stay, or wanting to stay but expecting to move.

BHPS, British Household Panel Survey.

For the historical housing circumstances, we pooled the responses from the last 10 years of the BHPS survey for each respondent, and transformed responses into a binary variable indicating whether or not each respondent had experienced the following issues at any point during that time.

### Control variables

We control for factors associated with housing and DNAmAge in our analysis. As noted above, previous analysis has found associations between DNAmAge measures and sex, nationality, education level, socioeconomic position, diet, cumulative stress, financial hardship, urban environments, socioeconomic position, body mass index and smoking.^3 4 8–12 30–34^ Importantly, pace of ageing increases as chronological age increases,^6^ making it necessary to control for chronological age. Many of these factors, such as socioeconomic position and age, are also associated with housing circumstances. These control variables are taken from the main survey prior to blood sample collection. Weighted proportions are shown in table 3.

**Table 3:** Weighted proportions for control variables, complete cases

Variable	Categories	Proportions
Sex	Male	0.469
	Female	0.531
Longstanding illness/disability	No	0.590
	Yes	0.410
Smoking status	Never smoked	0.399
	Former smoker	0.402
	Current smoker up to 10 p/d	0.064
	Current smoker 11–20 p/d	0.109
	Current smoker 21+ p/d	0.026
BMI	Under 18.5	0.005
	18.5 and below 25	0.294
	25 and below 30	0.415
	30 and below 40	0.249
	40 and above	0.038
Employment status	Employed	0.539
	Unemployed	0.034
	Retired	0.331
	Maternity/caring	0.061
	Student/other	0.008
	Long-term sick/disabled	0.028
Region	North East	0.054
	North West	0.116
	Yorkshire & Humber	0.123
	East Midlands	0.097
	West Midlands	0.090
	East of England	0.096
	London	0.075
	South East	0.117
	South West	0.111
	Wales	0.055
	Scotland	0.062
Highest qualification	Degree	0.166
	Other higher degree	0.100
	A-level, etc	0.198
	GCSE, etc	0.251
	Other qualification	0.130
	No qualification	0.154
Born in the UK	Yes	0.967
	No	0.033
Subjective financial situation	Living comfortably	0.275
	Doing alright	0.358
	Just about getting by	0.287
	Finding it quite difficult	0.058
	Finding it very difficult	0.021
Age-standardised income quartile	Lowest	0.269
	2	0.241
	3	0.240
	Highest	0.251
Age	Mean age of 55.4 years, Min. 28	

Values may not sum to 1 due to rounding.

BMI, body mass index; GCSE, General Certificate of Secondary Education.

**Figure 1:**
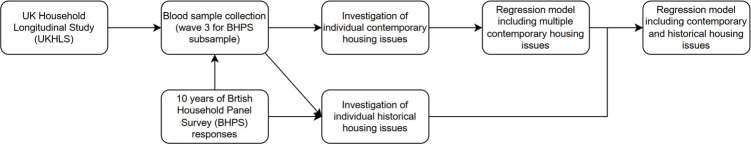
Flowchart showing study design.

We also account for blood cell composition and processing batch in our analysis.^4 21^ Analysis was conducted using Stata V.17. Weights were applied to account for unequal selection probabilities and stratification adjusted for using the SVY command. Analysis was run on complete cases (n=1312, or 92.4% of available sample, see online supplemental material tables A1–A3 and associated text for an overview of missing cases). We present linear associations between the individual housing variables and pace of ageing before presenting models which include multiple housing variables (see figure 1). Coefficients represent the speed of ageing in years: a coefficient of 0.5 would indicate a person ageing faster than expected: by 1.5 years, or 18 months, per year. Conversely, a negative coefficient would indicate slower ageing.

10.1136/jech-2023-220523.supp1 Supplementary data

## Results

Table 4 gives the results for the housing variables in the regression models predicting biological ageing measured using DunedinPoAm, full results can be found in online supplemental materials, table A4. A likelihood-ratio test indicated that a multilevel model nesting individuals within households did not provide better model fit than a regression model. The weighted mean of DunedinPoAm for the retained sample was 1.029 (SE 0.004, SD 0.079).

**Table 4:** Regression models predicting pace of ageing, housing variables

		Individual housing variable models	Model 1: Contemporary housing	Model 2: Contemporary and historical housing
Tenure (ref. Owned outright)	Owned with mortgage	0.008 (0.006)	0.007 (0.006)	0.009 (0.006)
	Social rent	0.013* (0.007)	0.002 (0.007)	0.000 (0.007)
	Private rent	0.047*** (0.010)	0.045*** (0.010)	0.046*** (0.011)
	Other	−0.068 (0.042)	−0.075^†^ (0.043)	−0.077 (0.046)
Receive housing benefit (ref. No)	Yes	0.007 (0.010)	−0.004 (0.009)	−0.008 (0.009)
Housing cost burden (ref. No)	Yes	0.015 (0.012)	0.000 (0.012)	−0.002 (0.013)
Property type (ref. Detached)	Semi-detached	0.008^†^ (0.005)	0.004 (0.005)	0.003 (0.005)
	Terrace	0.011* (0.005)	0.004 (0.006)	0.004 (0.006)
	Flat	0.033*** (0.008)	0.017^†^ (0.009)	0.016^†^ (0.008)
	Other	0.008 (0.019)	−0.001 (0.024)	−0.002 (0.025)
Housing payment arrears (ref. No)	Yes	0.017 (0.012)	0.009 (0.013)	0.004 (0.013)
Central heating (ref. Yes)	No	0.024** (0.010)	0.017 (0.011)	0.015 (0.012)
Overcrowding (ref. No)	Yes	0.014 (0.011)	0.012 (0.011)	0.009 (0.012)
Rural/urban (ref. Urban)	Rural	−0.006 (0.005)	−0.002 (0.005)	−0.006 (0.005)
Moving desire/ expectation (ref. Want to stay, expect to stay)	Want move, expect move	0.005 (0.008)	−0.009 (0.007)	−0.008 (0.007)
	Want stay, expect move	0.009 (0.012)	−0.015 (0.011)	−0.015 (0.012)
	Want move, expect stay	0.006 (0.005)	0.006 (0.005)	0.007 (0.005)
Ever report damp		0.009 (0.006)		0.007 (0.005)
Ever moving desire mismatch		0.001 (0.005)		0.000 (0.004)
Ever report space shortage		0.003 (0.005)		0.001 (0.005)
Ever report noise from neighbours		0.000 (0.005)		−0.006 (0.004)
Ever report street noise		−0.002 (0.005)		−0.009^†^ (0.005)
Ever report inadequate light		0.004 (0.005)		−0.001 (0.005)
Ever report inadequate heat		0.021*** (0.007)		0.013^†^ (0.007)
Ever report no central heating		0.008 (0.006)		−0.002 (0.006)
Ever report condensation		−0.003 (0.004)		−0.013** (0.005)
Ever report leaky roof		0.013** (0.006)		0.009 (0.007)
Ever report rot		0.006 (0.005)		0.001 (0.005)
Ever report pollution/environmental problems		0.010^†^ (0.005)		0.013* (0.005)
Ever report housing payment difficulties		0.015** (0.006)		−0.002 (0.007)
Ever report housing payment arrears		0.033*** (0.012)		0.025* (0.012)
Constant		Included	0.691** (0.241)	0.631** (0.226)

Models include blood cell composition and batch number (coefficients not shown).

†p<0.10; *p<0.05; **p<0.01; ***p<0.001, weighted n=901.

We first explore the relationships between DunedinPoAm and individual housing characteristics, controlling for cell count and batch numbers, as well as age, sex, longstanding illness and smoking status. When looking at housing circumstances in isolation, of the contemporary housing variables, only tenure, building type and central heating are significant at p<0.05. Renting, living in a flat or terrace and not having central heating are all associated with faster ageing relative to their reference categories. In terms of historical housing experiences, experiencing inadequate heating, a leaking roof, housing payment difficulties or housing payment arrears are significant.

Model 1 (column 2 of table 4) includes all contemporary housing experience predictor variables simultaneously. It shows statistically significantly faster biological ageing among private renters compared with those in outright ownership (0.045, p<0.001). With the inclusion of other housing variables in the model, the presence of central heating or living in a flat are no longer statistically significant.

In model 2, the historical housing variables are added. The coefficients for the contemporary variables are largely unchanged. Of the historical housing circumstances, reporting experiencing housing payment arrears was associated with faster ageing, whereas with the inclusion of other variables having experienced inadequate heating, leaking roof and payment difficulties were no longer statistically significant. Exploration of why historical, but not contemporary, experience of arrears is linked with biological ageing suggests that the result is driven by people who had reported multiple experiences of arrears, suggesting that repeated experience of arrears was the source of this finding. Having lived in a dwelling with pollution, grime or other environmental problems was also statistically significant. Interestingly, having reported condensation was associated with slower ageing. Further investigation suggests that variables for inadequate heating and damp are likely controlling out the negative effects associated with condensation, leaving the condensation variable to reflect the better housing and health of those able to adequately heat their homes resulting in condensation during cold weather.

For context, we find that the effect of living in a privately rented home on biological ageing is substantially greater than experiencing unemployment. The analysis suggests that living in a privately rented home (0.046 year) has an almost twofold greater impact on ageing than being unemployed (0.027 year), and that unemployment is similar in its effect on ageing to having experienced repeated rent arrears (0.025 year).

## Discussion

In this paper, we have explored the potential of biological ageing to shed light on the mechanisms underlying associations between housing and health. We find that renting privately, having experienced (repeated) payment arrears or living in a home affected by pollution is associated with faster ageing. Perhaps most notable, and robust (discussed below), is the faster ageing identified among private renters. Despite the stigmatisation of the tenure, social renting, with its lower cost and greater security of tenure, was not found to differ from outright ownership in terms of association with biological ageing once additional housing variables were included in the model. We posit that this may be related to the psychosocial benefits of additional security of tenure provided to social tenants. Our findings are consistent with existing understanding of epigenetic ageing, that ‘stress-induced acceleration of epigenetic aging may contribute to the long known link between psychological stress and aging-related disease phenotypes’^33^ and demonstrate a pathway by which housing circumstances can ‘get under the skin’ with real and significant consequences for health.

As with any analysis, there are several limitations to our findings. It should be noted that DNA methylation-derived measures of ageing are relatively new and therefore comparatively poorly understood, while the choice of DNAmAge measure can affect results.^4^ However, second-generation approaches have been found to outperform first-generation clocks,^35 36^ with DunedinPoAm, used here, outperforming other second-generation approaches.^32^ It is also a developing area. For example, an update to DunedinPoAm, DunedinPACE,^37^ has recently been developed but is not yet available in the UKHLS.

It is also important to note that although the broader survey data are longitudinal, the DNA methylation data used here are cross sectional. However, future collection rounds are planned which will enable greater understanding of housing circumstances over the life course and effects on biological ageing.^38^ The contemporary housing information from the UKHLS does not include any measures of housing quality, and so we must rely on historical circumstances of conditions and housing tenure. The 5-month gap between the main survey and the nurse survey/blood collection resulted in a loss to follow-up and means that housing circumstances may have been different when DNA methylation data were collected compared with when the survey responses were collected. The DNA methylation data also cover only white, European respondents, meaning that we are unable to consider the significant ethnic inequality in housing circumstances that exists in Britain. We are also unable to include children in our analysis. We also note that we have included a large number of variables in our analysis. We have included many control variables to take into account the findings of previous research and ensure that any associations with housing are accurate. We also include all potential housing characteristics to account for the wide-ranging and varied role of housing in health. Nonetheless, we recognise that this increases the risk of type I error. Applying the Bonferroni correction to account for the 23 housing variables results in a statistical significance threshold of p<0.00217, and of the housing variables only the coefficient for private renting is significant at this level. However, that the private renting coefficient remains significant at this very conservative level demonstrates the robustness of this finding.

Nonetheless, our analysis has produced some interesting and useful findings. Our finding that tenure is associated with faster ageing measured by DunedinPoAm at nearly half the rate of that associated with current smoking and twice that with obesity suggests that our results may have clinical significance. The significant role played by tenure and arrears in our analysis highlights the role of psychosocial factors linking housing with health via biological ageing. Research suggests that ‘epigenetic mechanisms are an important interface through which the body interprets and responds to stressful experiences’.^39^ It is likely that many of the physical housing conditions which were not significant in our analysis here influence health via a different mechanism. It is also possible that contemporary, rather than historical, housing conditions are important for pace of ageing. However, all these factors are policy amenable. What it means to be a private renter is not set in stone but dependent on policy decisions, which to date have prioritised owners and investors over renters. Policies to reduce the stress and uncertainty associated with private renting, such as ending ‘no-fault’ (Section 21) evictions, limiting rent increases and improving conditions (some of which have happened in parts of the UK since these data were collected) may go some way to reducing the negative impacts of private renting. Greater support with housing costs and restrictions on increasing housing costs may protect people from housing arrears and its health consequences. DNA methylation is reversible, suggesting that improving or changing the conditions for people with faster biological ageing can correct this, and health effects be mitigated or reversed.^5 29 31^ Therefore, housing policy changes can improve health. These findings are likely to be relevant to housing and health outside of Britain, particularly to countries with similar housing policies.

## Data Availability

Data are available in a public, open access repository. This work uses Understanding Society: Waves 1–11, 2009–2020 and Harmonised BHPS: Waves 1–18, 1991–2009 (SN: 6614) and Understanding Society: Waves 2 and 3 Nurse Health Assessment, 2010–2012 (SN: 7251). Colchester: These datasets are available to researchers through the UK Data Service (https://beta.ukdataservice.ac.uk/datacatalogue/series/series?id=2000053%23!/access-data) subject to registration.
