# Supplementary material for: Are housing circumstances associated with faster epigenetic ageing?
Source: J Epidemiol Community Health. 2023 Oct 10;78(1):40–6. doi: 10.1136/jech-2023-220523 (PMC10715511; doi:10.1136/jech-2023-220523)
Supplement: Supplementary data [file jech-2023-220523supp001.pdf]

Supplementary information

The subset of the UKHLS/BHPS sample used in our analysis is made representative of the British population through the application of weights calculated and provided by the UKHLS team. However, we present a complete case analysis which resulted in the dropping of 108 cases (7.6%). We briefly explore the impact of this here.

Table A1: Control variables, weighted differences across groups and missing cases

Variable	Statistical significance of difference between retained and dropped sample	Missing n
Sex	*p* = .926	0
Longstanding illness/disability	*p* = .021*,* 59.2% of dropped cases reported a longstanding illness compared to 41.0% of retained	0
Smoking status	*p* = .659	6
BMI	*p* = .601	30
Employment status	*p* = .012*,* those in the retained sample were less likely to be retired (33.1% v 61.5%) or long-term sick (6.26% v 2.76%) and less likely to be in the other categories	0
Region	*p* = .901	0
Highest qualification	*p* = .047*.* Education levels are higher among the retained sample, for example 16.6% have at least a degree, compared to 8.01% of the dropped sample.	9
Born in the UK	*p* = .567	23
Subjective financial situation	*p* = .565	0
Age-standardised income quartile	*p* = .944	1
Age	Mean age retained 55.4 years, mean age dropped 67.0 years *p* = .0001	0

These results show that the dropped and retained samples are broadly similar in terms of characteristics, with the exception of age – the retained sample is considerably younger than the dropped sample. It is likely that this difference in age drives the other significant differences – older people are more likely to have a longstanding illness or disability, to be retired, and have lower average education levels. The differences between these variables would not reach the threshold for statistical significance should a correction for multiple testing be applied.

Table A2: Contemporary housing variables, weighted differences across groups and missing cases

Variable	Statistical significance of difference between retained and dropped sample	Missing n
Tenure	*p* = .000*,* the retained sample were more likely to live in homes owned with a mortgage (38.7% v 19.5%) or private (6.93% v 1.44%) rented homes and less likely to live in social (13.8% v 28.9%) rented homes.	1
Building type	*p* = .001, semi-detached homes (34% v 43.7%) and flats (9.04% v 10.8%) were less common among the retained sample, detached (30.3% v 18.9%) and terraced (26.3% v 20.7%) were more common.	0
Receive housing benefit	*p* = .007*,* housing benefit receipt was lower among the retained sample (3.6% v 15.9%)	25
Has central heating	*p* = .986	3

Rural/urban	*p* = .633	0
Housing cost burden (spend over 1/3 of income on housing, income below median)	*p* = .000, housing cost burden was less common among the retained sample (2.7% v 24.2%)	9
Housing payment arrears (2+ months late with payment)	*p* = .458	3
Overcrowded (less than one bedroom per person/couple)	*p* = .312	0
Moving desires and expectations	*p* = .441	3

Differences in tenure across the samples likely reflect the age difference identified above, with renting, particularly private renting, less common among older populations in the UK. Likewise, the higher proportion of social renters among those in the dropped sample is likely in part due to the higher proportion of older people and people with long-term illness or disabilities living in this tenure. Flats are more common among social renters than in other tenures, potentially explaining their overrepresentation in the dropped sample. Although differences between the samples are significant, the small number of dropped cases means that the impact on the retained sample is minimal. For example, in the full, weighted sample 14.7% of participants live in social rented homes, this is 13.8% in the retained, weighted sample. It is therefore unlikely that these differences impacted the results of our analysis, particularly as we controlled for age which seems to be driving many of the significant differences. If anything, the younger and healthier nature of our retained sample is more likely to produce more conservative estimates for our housing variables, rather than exaggerate housing impacts on health.

Table A3: Historical housing variables, weighted differences across groups and missing cases

Variable	Statistical significance of difference between retained and dropped sample	Missing n
Damp walls, floors, foundation etc	*p* = .717	0
Shortage of space	*p* = .879	0
Noise from neighbours	*p* = .175	0
Other street noise	*p* = .863	0
Too dark, not enough light	*p* = .623	0
Lack of adequate heating facilities	*p* = .798	0
Condensation	*p* = .191	0
Leaky roof	*p* = .431	0
Rot in window frames or floors	*p* = .667	0
Pollution, grime or other environmental problems caused by traffic or industry	*p* = .479	0
Housing payment difficulties (problems paying for housing for over 1 year)	*p* = .508	0
Housing payment arrears	*p* = .482	0
Moving desires mismatch	*p* = .283	0
No central heating	*p* = .570	0

There were no significant differences in terms of historical housing variables between the retained and dropped sample.

Table A4: Full results of regression models predicting pace of ageing

		Individual housing variable models	Model 1: Contemporary housing	Model 2: Contemporary and historical housing
Age		included	0.000† (0.000)	0.000 (0.000)
Sex (ref. Male)	Female	included	-0.005 (0.005)	-0.006 (0.005)
Longstanding illness/disability (ref. No)	Yes	included	-0.007 (0.005)	-0.007 (0.005)
Smoking status (ref. never smoked)	Former smoker	included	0.022*** (0.005)	0.021*** (0.005)
	Current smoker (up to 10 per day)	included	0.085*** (0.014)	0.078*** (0.012)
	Current smoker (11-20 per day)	included	0.127*** (0.010)	0.125*** (0.009)
	Current smoker (21+ per day)	included	0.144*** (0.013)	0.144*** (0.012)
BMI (ref. 18.5-24.9)	Under 18.5	included	-0.041* (0.020)	-0.044* (0.021)
	25 and below 30	included	0.008 (0.005)	0.008 (0.005)
	30 and below 40	included	0.022*** (0.006)	0.020** (0.006)
	40 and above	included	0.022† (0.012)	0.014 (0.012)
Employment status (ref. Employed)	Unemployed	-	0.023* 0.012	0.027* 0.012
	Retired	-	0.007 (0.007)	0.008 (0.007)
	Maternity/caring	-	0.006 (0.011)	0.013 (0.010)
	Student/other	-	-0.019† (0.011)	-0.025* (0.012)
	Long-term sick/disabled	-	-0.009 (0.013)	-0.013 (0.013)
Region (ref. North East)	North West	-	-0.018 (0.011)	-0.011 (0.011)
	Yorkshire & Humber	-	-0.017 (0.011)	-0.012 (0.011)
	East Midlands	-	-0.020† (0.011)	-0.016 (0.012)
	West Midlands	-	-0.010 (0.011)	-0.008 (0.011)
	East of England	-	-0.014 (0.012)	-0.011 (0.012)
	London	-	-0.007 (0.013)	-0.008 (0.013)
	South East	-	-0.025* (0.010)	-0.017 (0.011)
	South West	-	-0.016 (0.011)	-0.011 (0.011)
	Wales	-	-0.026† (0.014)	-0.024† (0.014)
	Scotland	-	-0.018 (0.011)	-0.015 (0.012)

Highest qualification (ref. Degree)	Other higher degree	-	0.012† (0.007)	0.013† (0.007)
	A-level etc	-	0.016* (0.006)	0.017** (0.006)
	GCSE etc	-	0.015* (0.006)	0.015* (0.006)
	Other qualification	-	0.003 (0.009)	0.004 (0.009)
	No qualification	-	0.017* (0.008)	0.021* (0.009)
Born in the UK (ref. Yes)	No	-	-0.017† (0.009)	-0.013 (0.009)
Subjective financial situation (ref. Living comfortably)	Doing alright	-	-0.005 (0.005)	-0.003 (0.006)
	Just about getting by	-	-0.004 (0.007)	-0.004 (0.007)
	Finding it quite difficult	-	0.004 (0.011)	0.003 (0.012)
	Finding it very difficult	-	0.015 (0.015)	0.014 (0.016)
Age-standardised income quartile (ref. Lowest)	2	-	0.004 (0.005)	0.005 (0.005)
	3	-	-0.001 (0.006)	-0.002 (0.005)
	Highest	-	-0.003 (0.008)	-0.005 (0.007)
Tenure (ref. Owned outright)	Owned with mortgage	0.008 (0.006)	0.007 (0.006)	0.009 (0.006)
	Social rent	0.013* (0.007)	0.002 (0.007)	0.000 (0.007)
	Private rent	0.047*** (0.010)	0.045*** (0.010)	0.046*** (0.011)
	Other	-0.068 (0.042)	-0.075† (0.043)	-0.077 (0.046)
Receive housing benefit (ref. No)	Yes	0.007 (0.010)	-0.004 (0.009)	-0.008 (0.009)
Housing cost burden (ref. No)	Yes	0.015 (0.012)	0.000 (0.012)	-0.002 (0.013)
Property type (ref. Detached)	Semi-detached	0.008† (0.005)	0.004 (0.005)	0.003 (0.005)
	Terrace	0.011* (0.005)	0.004 (0.006)	0.004 (0.006)
	Flat	0.033*** (0.008)	0.017† (0.009)	0.016† (0.008)
	Other	0.008 (0.019)	-0.001 (0.024)	-0.002 (0.025)
Housing payment arrears (ref. No)	Yes	0.017 (0.012)	0.009 (0.013)	0.004 (0.013)
Central heating (ref. Yes)	No	0.024** (0.010)	0.017 (0.011)	0.015 (0.012)
Overcrowding (ref. No)	Yes	0.014 (0.011)	0.012 (0.011)	0.009 (0.012)
Rural/urban (ref. Urban)	Rural	-0.006 (0.005)	-0.002 (0.005)	-0.006 (0.005)

Moving desire/ expectation (ref. Want to stay, expect to stay)	Want move, expect move	0.005 (0.008)	-0.009 (0.007)	-0.008 (0.007)
	Want stay, expect move	0.009 (0.012)	-0.015 (0.011)	-0.015 (0.012)
	Want move, expect stay	0.006 (0.005)	0.006 (0.005)	0.007 (0.005)
Ever report damp		0.009 (0.006)		0.007 (0.005)
Ever moving desire mismatch		0.001 (0.005)		0.000 (0.004)
Ever report space shortage		0.003 (0.005)		0.001 (0.005)
Ever report noise from neighbours		0.000 (0.005)		-0.006 (0.004)
Ever report street noise		-0.002 (0.005)		-0.009† (0.005)
Ever report inadequate light		0.004 (0.005)		-0.001 (0.005)
Ever report inadequate heat		0.021*** (0.007)		0.013† (0.007)
Ever report no central heating		0.008 (0.006)		-0.002 (0.006)
Ever report condensation		-0.003 (0.004)		-0.013** (0.005)
Ever report leaky roof		0.013** (0.006)		0.009 (0.007)
Ever report rot		0.006 (0.005)		0.001 (0.005)
Ever report pollution/environmental problems		0.010† (0.005)		0.013* (0.005)
Ever report housing payment difficulties		0.015** (0.006)		-0.002 (0.007)
Ever report housing payment arrears		0.033*** (0.012)		0.025* (0.012)
Constant		Included	0.691** (0.241)	0.631** (0.226)
Models include blood cell composition and batch number (coefficients not shown). † $p < .10$, * $p < 0.05$, ** $p < 0.01$, *** $p < 0.001$, weighted n=901				
